# Defining and measuring varus thrust in knee osteoarthritis: A scoping review of current evidence and challenges

**DOI:** 10.1016/j.ocarto.2025.100683

**Published:** 2025-09-13

**Authors:** Vincenzo E. Di Bacco, Zaryan Masood, Joshua A.J. Keogh, Matthew C. Ruder, Fatima Gafoor, Jenny Wu, Yalda Azari, Eseoghene Orogun, Dylan Kobsar

**Affiliations:** a Department of Kinesiology, Faculty of Science, McMaster University, Hamilton, Canada; b School of Biomedical Engineering, Faculty of Engineering, McMaster University, Hamilton, Canada

**Keywords:** Gait analysis, Wearable sensors, Visual assessment, Optical motion capture, Kinematics

## Abstract

**Objective:**

This scoping review investigated the definitions, assessment methods, and current applications of varus thrust (VT) in knee osteoarthritis (OA).

**Methods:**

Five databases (MEDLINE, EMBASE, CINAHL, SPORTDiscus, and Web of Science Core Collection) were searched in this scoping review for studies assessing VT during walking in adults with knee OA using the terms “varus” and “lateral” in proximity to thrust. Data were extracted and categorized by study characteristics (OA sample, publication year, design, and aim) and VT assessment protocol (method and definition).

**Results:**

A total of 63 studies were included, examining 12,569 individuals with knee OA using visual (*n* ​= ​24), optical motion capture (*n* ​= ​27), or inertial/wearable sensor (*n* ​= ​19) methods. Designs included prospective, experimental, cross-sectional, and case series. VT was most often assessed to examine disease severity, progression, surgical outcomes, and symptom associations. Visual VT was commonly defined as dynamic worsening or abrupt onset of varus alignment during weight acceptance. Optical motion capture commonly measured VT as frontal plane knee excursion from foot contact to mid-stance, while inertial methods typically used peak lateral tibial acceleration or angular velocity.

**Conclusion:**

Despite growing research interest in VT, inconsistent definitions and measurement protocols limit comparability across studies and hinder broader adoption. Greater standardization and validation are needed to clarify its potential clinical utility in knee OA.

## 1 Introduction

Biomechanical factors contribute to both the structural and symptomatic aspects of knee osteoarthritis (OA) [1,2]. Frontal plane knee loading, typically inferred from the knee adduction moment (KAM), has received substantial attention [3,4]. However, KAM requires motion capture and force plates, limiting feasibility in many settings. As a result, there is growing interest in accessible markers of dynamic joint loading to improve OA monitoring and management.

Varus thrust (VT) has been proposed as a kinematic marker that may serve as a proxy for medial knee loading. It has been defined as an abrupt increase in varus or lateral knee motion during the weight acceptance phase of gait [5], though definitions vary and a clear consensus is lacking. Although VT is not a kinetic measure, its presence has been associated with higher KAM values [5,6], supporting its relevance as an indirect indicator of medial knee loading. As a brief, observable gait event, VT is often assessed visually [7]; however, this method is limited by uncertain reliability and dependent on evaluator experience. These limitations have driven interest in more objective methods using motion capture systems [8] and wearable inertial sensors [9].

VT has been studied in relation to disease severity [10,11], progression [5], and responses to rehabilitation and treatment [12,13]. Despite this growing interest, both the definition and assessment of VT remain inconsistent. This scoping review aims to consolidate the fragmented literature and offer a cohesive interpretation of VT's role in gait assessment for individuals with knee OA. Specifically, we sought to (i) map the varying operational definitions and measurement protocols used to quantify VT; (ii) identify common study designs and applications; and (iii) highlight gaps in the current literature to inform future research directions.

## 2 Methods

### 2.1 Methodological framework

A scoping review methodological framework was selected to address the knowledge gaps surrounding the assessment and application of VT, and to align with the exploratory nature of the research objectives [14,15]. The review was conducted and reported in accordance with the Preferred Reporting Items for Systematic Reviews and Meta-Analyses extension for Scoping Reviews (PRISMA-ScR) [16].

### 2.2 Eligibility criteria

Studies were eligible for inclusion if they examined VT during walking in adults with knee OA. Studies were excluded if they: (i) did not include adults with knee OA; (ii) did not assess VT in the context of walking; (iii) were conference abstracts, systematic reviews, or a meta-analysis; or (iv) were not published in English.

### 2.3 Search strategy

An electronic search was conducted in MEDLINE, EMBASE, CINAHL, SPORTDiscus, and Web of Science Core Collection. The search strategy included the terms “varus” and “lateral” in proximity to the term “thrust” (e.g., adj5, near/5, or N5), allowing for variations such as “varus knee thrust”. This targeted approached was designed to capture studies specifically focused on the concept of VT. No additional filters or restrictions were applied.

### 2.4 Study selection

The systematic search was performed by DK on July 7th, 2025, and all results were imported into Covidence for screening. Duplicate records were automatically flagged by Covidence and manually verified by DK prior to removal. Title and abstract screening, followed by full-text review, was conducted independently by at least two reviewers using the predefined eligibility criteria. Discrepancies were resolved through discussion, with DK acting as a third reviewer when consensus could not be reached. Reference lists of all included studies were also hand-searched to identify any additional relevant articles.

### 2.5 Data charting and synthesis

Following full-text screening, data were extracted by two authors and verified for accuracy by the primary researcher (DK) using Covidence. Extracted data were grouped into two broad categories: study characteristics and VT assessment protocol. Study characteristics included OA sample details, publication year, study design, and study aim. VT assessment protocol captured the measurement system used and the operational definition of VT. These data were synthesized by categorizing study design and measurement systems, while summarizing study aims and VT definitions to support the narrative synthesis. Studies contributing to multiple themes were documented accordingly. As a result, the number of reported entries may exceed the number of included studies due to multiple outcomes being assessed within individual studies.

## 3 Results

**Fig. 1:**
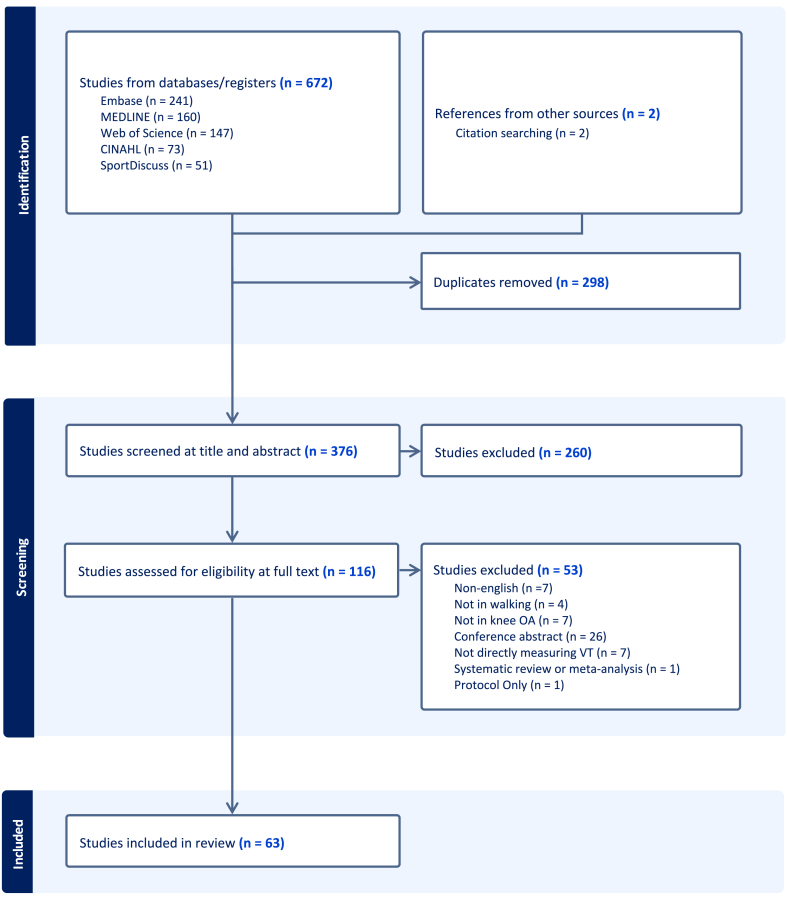
PRISMA flowchart of the scoping review selection process.

**Fig. 2:**
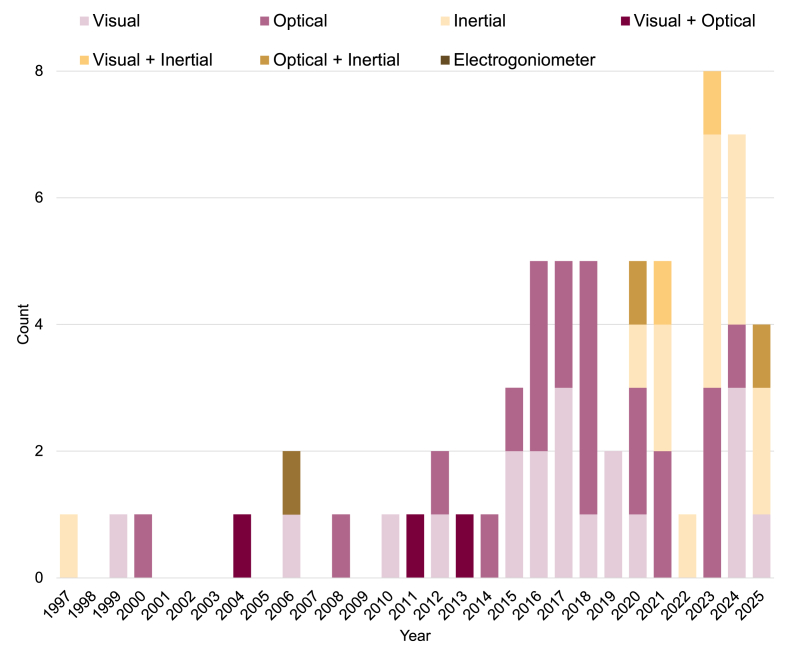
Varus thrust publication count over time, differentiated by assessment method.

The search strategy identified 672 articles. After removing 298 duplicates, 376 studies remained for title and abstract screening. Of these, 116 were passed to full-text screening, with these further reduced to 63 studies included in the final scoping review (Fig. 1). The PRISMA flow diagram (Fig. 1) outlines the screening process and reasons for full-text exclusions. Extracted data from the studies, including study design, aim, and VT assessment methods are summarized in Table 1. The publication count per year for each VT assessment method is summarized in Fig. 2, outlining the increasing popularity of VT gait in knee OA research over time.

**Table 1:** Study design, sample, and varus thrust assessment summary of the 63 included studies.

Author	Year	Design	Aim	VT Measure	VT Definition	KOA Sample
Aoda et al. [35]	2006	Cross-sectional: Between groups (severity)	Identify factors associated with radiographic severity	Visual assessment	The acute adduction of the knee joint at the early stance of the gait cycle	688^DB, S^
Chang et al. [10]	2010	Cross-sectional: Between groups (severity)	Determine the frequency of varus and valgus thrust in African Americans and Caucasians, and to identify factors associated with VT presence	Visual assessment	Dynamic worsening or abrupt onset of varus alignment as the limb accepted weight, with a return to less varus alignment during lift-off and the swing phase of gait	2026^DB, S^
Omori et al. [59]	2016	Cross-sectional: Between groups (severity) and correlation	Identify mechanical factors (e.g., VT) related to knee OA severity and pain	Visual assessment	Acute adduction of the knee joint during the early stance phase of the gait cycle, classified into three groups: definitely present, possibly present, or definitely absent	699^DB, S^
Fukutani et al. [42]	2016	Cross-sectional: Correlation	Investigate the association between VT and pain, stiffness, and activities of daily living	Visual assessment	Dynamic worsening or an abrupt onset of varus alignment as the limb accepted weight, with a return to less varus alignment during lift-off and the swing phase of the gait	284
Ohi et al. [58]	2018	Cross-sectional: Correlation	Examine the association between 3D static foot posture and VT in medial knee OA patients	Visual assessment	Dynamic worsening or an abrupt onset of varus alignment as the limb accepts weight, with a return to reduced varus alignment	88
Lo et al. [51]	2012	Cross-sectional: Correlation	Investigate associations of VT and varus static alignment with pain in patients with knee OA	Visual assessment	The first appearance of varus or abrupt worsening of existing varus while the limb is bearing weight during ambulation, with a return of the limb to a less varus alignment during the swing, or non-weight-bearing, phase of gait	82^S^
Iijima et al. [45]	2015	Cross-sectional: Correlation	Investigate the association between knee pain during gait and 4 clinical phenotypes based on static varus alignment and VT in patients with medial KOA	Visual assessment	Dynamic worsening or abrupt onset of varus alignment in the stance phase, with a return to less varus alignment in the swing phase during the gait cycle	266
Iijima et al. [44]	2017	Cross-sectional: Correlation	Investigate the association of VT during gait with patellofemoral OA grade, alignment, and pain	Visual assessment	Two investigators classified VT as “definitely present” for the participants, to determine whether they would be classified in a group with or without definite varus thrust	171^S^
Iijima et al. [43]	2020	Cross-sectional: Correlation	Determine the association between VT and low back pain in patients with knee OA	Visual assessment	Lateral movement of the tibial tuberosity relative to hip and ankle joints, independent from hip external rotation and/or knee flexion motion	205
Bennell et al. [17]	2015	Experimental: Exercise	Investigate whether biomechanics influence changes in pain and physical characteristics with exercise in patients with medial KOA and varus malalignment	Visual assessment	Visually observable dynamic worsening or abrupt onset of varus malalignment in OA knee during the stance phase of the gait cycle, with a return to less varus alignment during the lift-off and swing phases of the gait cycle	100^S^
Iijima et al. [22]	2024	Experimental: Exercise	To assess the influence of VT on the therapeutic effect of home-based unsupervised quadriceps exercise on knee pain and to identify mechanisms behind the impact of VT on the exercise, in individuals with or at increased risk of KOA	Visual assessment	Two physical therapists classified VT as “definitely present, possible present, or definitely absent” for the participants, to determine whether they would be classified in a group with VT (definitely or possibly present) or without VT (definitely absent)	50
D'Souza et al. [19]	2024	Experimental: Gait retraining	Investigate the effects of 3 gait retraining interventions (toe-in, toe-out, and placebo gait) on proxy measures of medial knee load (e.g., KAM, VT)	Visual assessment	The abrupt lateral movement of the knee during the stance phase of gait, with a return to a less varus alignment during swing, rated as either “present” or “absent”	9
Iijima et al. [21]	2019	Prospective: Exercise	Determine if there is an interaction effect between ambulatory physical activity and VT on knee pain in patients with KOA	Visual assessment	Two clinicians classified VT as “definitely present” for the participants, to determine whether they would be classified in a group with or without definite varus thrust	207
Sharma et al. [28]	2017	Prospective: Progression	Determine if VT is associated with incident and progressive KOA	Visual assessment	Broadly as the bowing out or dynamic worsening of varus alignment during gait	2610^DB, S^
Wink et al. [34]	2017	Prospective: Progression	Determine association of VT to incident and worsening medial tibiofemoral cartilage damage in older adults with or at risk for KOA	Visual assessment	High-speed recordings with markers over patellae and tibial tuberosities to assess the dynamic worsening or abrupt onset of varus alignment during weight acceptance phase of gait. VT was rated then on a Likert-type scale before being dichotomized to present or absent	1007^DB, S^
Wink et al. [33]	2019	Prospective: Progression	Investigate the association of VT during gait to the odds of worsening WOMAC knee pain in older adults with or at risk of KOA	Visual assessment	Dynamic worsening or abrupt onset of varus alignment during the weight-acceptance phase of the gait cycle, with a return to more neutral alignment during the lift-off and swing phases of the gait cycle	1623^DB, S^
Naudie et al. [7]	1999	Prospective: Surgery	Examine the trajectory of patients with knee OA that underwent high tibial osteotomies and the possible relationship baseline factors such as VT	Visual assessment	Simply defined as “presence of a lateral tibial thrust”	85
Zorzi et al. [69]	2024	Case Series (Surgery)	To evaluate knee arthroplasty and sliding osteotomy in correcting rigid valgus deformity and other clinical features like VT	Visual assessment	Simply defined as “presence of varus thrust during gait”	19
Haider et al. [70]	2025	Case study: Surgery	To highlight the risk of late implant failure in unrestricted kinematically aligned TKA	Visual assessment	Simply defined as “bow leg deformity” or “severe varus thrust during walking"	1
Paterson et al. [60]	2017	Cross-sectional: Between groups (sex)	Determine sex-based differences and influence of obesity status on gait in patients with severe KOA	3D optical kinematics of knee	The largest, most abrupt frontal plane knee movement (angle) in either the varus or valgus direction during the first 30 ​% of stance that coincides with peak knee angular velocity	34
Sosdian et al. [11]	2016	Cross-sectional: Between groups (OA vs. controls)	Determine differences in biomechanical parameters and direction of VT in patients awaiting TKA vs. asymptomatic controls	3D optical kinematics of knee	The largest, most abrupt frontal plane knee movement (angle) in either the varus or valgus direction during the first 30 ​% of stance that coincides with peak knee angular velocity	44
Mezghani et al. [54]	2018	Cross-sectional: Between groups (OA vs. controls)	Use 3D knee kinematic data to classify knee OA and healthy control gait	3D optical kinematics of knee	Varus thrust during loading (1–30 ​%) as reported from proprietary KneeKG software	40
Mahmoudian et al. [52]	2016	Cross-sectional: Between groups (severity, OA vs. controls)	Investigate the relationship between VT and the knee abduction moment in early OA, established OA, and healthy controls	3D optical kinematics of knee	The difference between the knee adduction angle at heel strike and the first maximum knee adduction angle (excursion) during the stance phase of the gait cycle	47
Leporace et al. [50]	2021	Cross-sectional: Between groups (clustering)	Determine whether knee kinematics can identify different gait profiles in knee OA patients using machine learning	3D optical kinematics of knee	Gait patterns broadly characterized as varus thrust based on interpretation of waveforms via principal component analysis	42^S^
Espinosa et al. [40]	2020	Cross-sectional: Between groups (OA vs. controls), plus correlations	Investigate relationships between VT and isokinetic strength knee extensors and flexors in people with and without OA	3D optical kinematics of knee	Knee adduction excursion and peak adduction velocity, both in the first half of stance	28
Takigami et al. [8]	2000	Cross-sectional: Between groups (severity) plus correlation	Assess the relationship between knee OA symptoms and the angular velocity of VT	3D optical kinematics of knee	Mean angular velocity between femur and tibia from heel strike to foot flat using three markers at the hip, knee, and ankle	28
Kuroyanagi et al. [6]	2012	Cross-sectional: Between groups (severity) plus correlation	Examine the relationship of VT with knee OA severity, and other dynamic and static evaluations	3D optical kinematics of knee	The difference in frontal plane angle from heel strike to stance peak (excursion), as defined by a single marker at the hip, knee, and ankle	32
Dixon et al. [39]	2018	Cross-sectional: Between groups (severity) plus correlation	Examine correlation between muscle co-contraction and VT and the modulation by disease severity of history of knee ligament rupture	3D optical kinematics of knee	The mean absolute difference between knee adduction angle at heel strike and the maximum knee adduction angle (excursion) between heel strike and the end of mid-stance across five gait trials	42
Ishii et al. [47]	2023	Cross-sectional: Correlation to meniscal extrusion and between groups (OA vs. controls)	To investigate the behavior of the lateral meniscus during walking using dynamic ultrasonographic evaluation and gait metrics	3D optical kinematics of knee	The difference (excursion) in varus angle to the stance phase (20 ​% between the maximum and minimum)	16
Ishii et al. [46]	2023	Cross-sectional: Correlation to meniscal extrusion and between groups (OA vs. controls)	To investigate the pattern of meniscal behaviour during walking and its association with limb biomechanics	3D optical kinematics of knee	The difference in knee angle between the varus and valgus (excursion) occurring in the early stance phase of the gait cycle	55
Azuma et al. [36]	2023	Cross-sectional: Correlation	To determine the association between clinical symptoms and VT following HTO	3D optical kinematics of knee	The difference between the maximum and minimum change (excursion) in the knee varus angle during the early stance phase	54
van der Esch [65]	2008	Cross-sectional: Correlation	Assess the relationship between knee varus-valgus motion and both muscle strength and functional ability	3D optical kinematics of knee	Difference between peak excursion in the varus vs. valgus direction at midstance when peak ground reaction force was reached	63
Fukaya et al. [41]	2015	Cross-sectional: Correlation	Examine frontal and horizontal plane kinematics in patients with severe knee OA	3D optical kinematics of knee	Difference in knee varus angle from initial contact to loading response (12 ​% of gait)	12
Hall et al. [12]	2018	Experimental: Exercise	Determine if the effects of exercise on pain are modulated by frontal plane kinematics	3D optical kinematics of knee	Peak varus angle, frontal plane excursion, and peak angular varus velocity in first 40 ​% of stance	323^DB, S^
Mahmoudian et al. [24]	2017	Prospective: Progression	Evaluate the relationship between frontal plane static and dynamic knee alignment with structural and clinical characteristics of KOA between early and established KOA	3D optical kinematics of knee	Frontal plane knee alignment calculated as the difference between knee adduction angle at heel strike and the first maximum knee adduction angle during the stance phase of the gait cycle	47
Shimada et al. [30]	2016	Prospective: Surgery	Identify the presence of VT 1 year post-operatively	3D optical kinematics of knee	The varus-valgus angular displacement (maximum – minimum varus) during the loading response phase, defined as the first 10 ​% of the gait cycle	15
Paterson et al. [26]	2018	Prospective: Surgery	Assess changes in gait following TKA and the influence of sex and obesity	3D optical kinematics of knee	Varus (+) or valgus (−) movement with the greatest angular excursion in the first 30 ​% of stance; while also referencing Sosdian et el. [11]	43
Paterson et al. [13]	2020	Prospective: Surgery	Evaluate changes in frontal and sagittal plane kinematics following TKA and the influence of sex and obesity	3D optical kinematics of knee	Simply as Varus-valgus thrust excursion while referencing Sosdian et el. [11]	78^S^
Mezghani et al. [25]	2021	Q-Exp: Exercise	Assess baseline gait parameters for predicting response to exercise	3D optical kinematics of knee	Maximum knee adduction angle between 10 and 30 ​% of the gait cycle minus the value at 10 ​% of gait cycle	221^S^
Deie et al. [18]	2014	Q-Exp: Surgery	Investigate changes following TKA in clinical symptoms and knee joint biomechanics during gait	3D optical kinematics of knee	Difference between maximum and minimum knee varus angles during the loading phase (0–10 ​%)	21
Shimada et al. [29]	2024	Q-Exp: Muscle stimulation	To investigate whether modification of vastus medialis activity can delay the varus thrust	3D optical kinematics of knee	The difference between the minimum knee varus angle from the maximum varus angle in the load response period of gait	10
Tsurumiya et al. [64]	2021	Cross-sectional: Between groups (severity, OA vs. controls)	Assess gait and VT of asymptomatic participants and knee OA patients based on progression status	Inertial sensor (proximal anterior tibia, plus thigh and pelvis)	First lateral acceleration peak and first angular velocity peak assessed independently. If no peak occurred, the largest value between 0 and 20 ​% stance was used	15
Misu et al. [56]	2022	Cross-sectional: Between groups (OA vs. controls) plus reliability	This study aimed to examine the reliability of VT and whether VT could distinguish knee OA patients with varus thrust from healthy adults	Inertial sensor (proximal anteromedial tibia and lateral distal thigh)	Root mean square of mediolateral accelerations during the stance phase, for both thigh and shank sensors	16
Ishii et al. [48]	2020	Cross-sectional: Between groups (severity) plus correlation	Examine correlation between VT and meniscus excursion and to determine whether differences existed based on early and severe KOA	Inertial sensor (tibial tuberosity)	Average lateral acceleration peak from five continuous strides	44
Iwama et al. [49]	2021	Cross-sectional: Correlation	Estimate knee adduction moment (KAM) using a single IMU and validating that during gait against KAM obtained during conventional motion capture	Inertial sensor (6 locations: anterior mid-thighs and proximal tibias, plus pelvis and chest)	VT was defined as the peak-to-peak difference of acceleration in the lateral/medial axis immediately after heel contact from sensors	22
Wada et al. [66]	2023	Cross-sectional: Correlation	To investigate the effect of repetitive physical activity on knee joint laxity	Inertial sensor (lateral mid-tibia and distal thigh)	The magnitude of mediolateral accelerations based on the range from positive and negative peaks	68
Misu et al. [55]	2023	Cross-Sectional: Correlation	To investigate the association between VT and patient-reported outcome measures in OA	Inertial sensor (anterior tibia)	Calculated a VT-index by dividing RMS of ML accelerations in the first half of stance with RMS of angular velocity in swing phase	70
Taniguchi et al. [61]	2024	Cross-sectional: Correlation	To clarify the relationship between fear-avoidance beliefs and co-contraction during gait and stair climbing in people with KOA	Inertial sensor (proximal anterior tibia, with additional sensor on dorsum of foot)	Peak lateral acceleration in early stance phase	20
Makino et al. [53]	2025	Cross-sectional: Correlation	To investigate the relationship between foot alignment and flexibility and VT during gait in patients with KOA, and to develop a physiotherapy approach for the foot to decrease VT	Inertial sensor (tibial tuberosity, with an additional sensor on foot)	Calculated a VT-index by dividing RMS of ML accelerations in the first half of stance with RMS of angular velocity in swing phase	20
Wada et al. [32]	2025	Prospective: Surgery	To determine whether VT differs between OA patients and healthy, control subjects, and whether this is changed with HTO surgery	Inertial sensor (lateral thigh and lateral tibia)	Range between the positive peak and negative peak in the frontal plane accelerations of the thigh relative to the shank	60
Sato et al. [27]	2024	Prospective: Surgery	To investigate the relationship between clinical outcomes and lateral thrust before and after unicompartmental knee arthroplasty	Inertial sensor (anterior thigh and anteromedial tibia)	Peak varus angular velocity	8
Ogata et al. [9]	1997	Q-Exp: Insoles	Quantify VT in healthy and OA knees with and without insoles and to determine the effects of the insoles on levels of pain	Inertial sensor (tibial tuberosity)	First peak mediolateral acceleration value defined as VT if motion was lateral, and valgus thrust if the first peak displayed medial motion	48
Ishii et al. [23]	2023	Q-Exp: Insoles	To investigate the effect of lateral wedge insole on gait in knee OA patients over 3 weeks	Inertial sensor (tibial tuberosity, with an additional sensor on foot)	VT was measured as the lateral acceleration peak on the shank sensor up to the first 10 ​% of the gait cycle	15
Edo et al. [20]	2023	Q-Exp: Insole	To investigate the effect of lateral wedge insole on gait in knee OA patients in the same session	Inertial sensor (proximal lateral and anterior tibia, with a third on the heel)	VT was measured as the peak value of the lateral acceleration during stance from the shank sensor on the fibular head	8
Tanaka et al. [68]	2024	Case Study: Insole	Examine the effect of lateral wedge insoles on VT and its potential relation to bone marrow lesions	Inertial sensor (tibial tuberosity and dorsal foot)	Peal acceleration immediately after contact	1
Tsukamoto et al. [62]	2023	Cross-sectional: Between groups (severity) plus validity	The purpose was to develop a novel frontal plane classification system using mediolateral accelerations on the thigh and shank VT that related to severity	Inertial sensors (proximal anterior tibia and mid-thigh) and visual assessment	VT was defined by the maximum varus angle with the largest peak knee angular velocity during the first 30 ​% of the stance phase. Visually defined as the sudden deterioration of knee alignment laterally during the stance phase and identified simply as “the presence of clearly visible varus thrust.”	69
Chang et al. [37]	2013	Cross-sectional: Correlation	Determine if visually observed VT is associated with greater knee varus dynamic movement (e.g., peak knee varus angular velocity)	3D optical kinematics of knee and visual assessment	No operational definition other than the visual determination of the presence of varus thrust which was reported on a Likert Scale of “very confident, somewhat confident, not very confident, or not at all confident”	236^DB, S^
Costello et al. [38]	2020	Cross-sectional: Validity	Compare VT quantification between inertial sensor and optical motion capture	3D optical kinematics of knee and inertial sensor on lateral mid-thigh and mid-tibia	Difference in adduction angle between initial contact and maximum in first half of stance, and peak knee angular velocity between initial contact and midstance from sensors	26
Tsukamoto et al. [63]	2021	Cross-sectional: Validity	Clarify the diagnostic accuracy of VT measurement, using IMUs	Visual assessment and inertial sensor (anterior mid-thigh and proximal anterior tibia)	Lateral dynamic worsening was visually rated on a Likert-type scale, then dichotomized into present, absent, or ambiguous. Sensors defined VT as the peak varus angle and angular velocity from the inertial sensors	49
Murro et al. [57]	2025	Cross-sectional: Validity plus between groups (OA vs. controls)	To compare estimates of VT between motion capture and inertial measurement unit data in adults who were asymptomatic or who had KOA	3D optical kinematics and inertial sensor (midpoint of lateral thigh and shank, with an additional sensor on the dorsum of the foot)	Motion capture defined as peak knee adduction angular velocity during midstance (10–30 ​%). Sensor defined as peak angular adduction velocities of the thigh, shank, and knee (by subtracting thigh and shank angular velocities) during midstance (10–30 ​%)	17
Chang et al. [5]	2004	Prospective: Progression	Determine whether the presence of VT at baseline increases the risk of progression or medial tibiofemoral OA	Visual assessment and 3D optical kinematics of knee (for a subset of participants; *n* ​= ​64)	The abrupt first appearance of varus or worsening of existing varus, while the limb is bearing weight during ambulation, with return to a less varus alignment during the non-weight-bearing phase of gait. Optical (for force platform) defined VT as the maximum adduction moment peak	222^DB, S^
Takemae et al. [31]	2006	Q-Exp: Surgery	Evaluate the change in 3D knee motion before and after high tibial osteotomy	Other Sensor: Electrogoniometer	VT was defined as rapid adduction during the early stance phase of the gait cycle	19
Hunt et al. [67]	2011	Case study (gait retraining, insole)	Describe biomechanics of VT during gait and to evaluate the biomechanical effects of gait-related interventions aimed at minimizing VT in a KOA patient	3D optical kinematics of knee and visual assessment	Visually identified as a noticeable lateral displacement of the right knee soon after initial foot contact, and optically as a knee adduction peak at 20 ​% stance	1

Abbreviations: VT ​= ​varus thrust, TKA ​= ​Total Knee Arthroplasty, OA = Osteoarthritis, KOA = Knee OA, ^DB^ = Patient data obtained from database; ^S^ = Secondary analysis of previously collected data, Experimental ​= ​RCT (control group, randomization), Q-Exp ​= ​Quasi-experimental (intervention may or may not include a control group or randomization; often within-subject design).

### 3.1 Study designs and applications

**Fig. 3:**
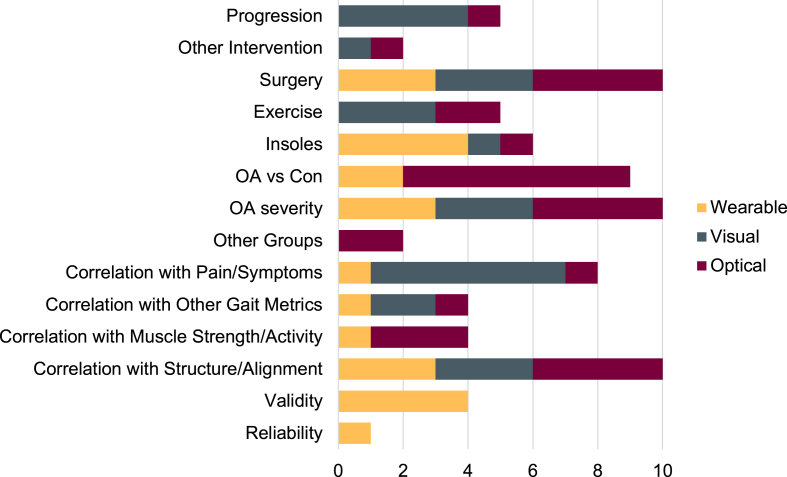
Varus thrust applications across different experimental protocols. OA = Osteoarthritis; Con ​= ​control group.

Among the 63 included studies, we identified 12 prospective designs, 11 using experimental or quasi-experimental approaches [5,7,9,12,13,[17], [18], [19], [20], [21], [22], [23], [24], [25], [26], [27], [28], [29], [30], [31], [32], [33], [34]], 36 cross-sectional studies [6,8,10,11,[35], [36], [37], [38], [39], [40], [41], [42], [43], [44], [45], [46], [47], [48], [49], [50], [51], [52], [53], [54], [55], [56], [57], [58], [59], [60], [61], [62], [63], [64], [65], [66]], and 4 case studies or case series [[67], [68], [69], [70]]. These studies leveraged VT to explore disease progression, treatment response, symptom associations, and group comparisons, among other aims. Further detail on study design and application is provided below, as well as in Table 1 and visually summarized in Fig. 3.

Prospective and experimental designs were primarily used to examine VT in relation to: (i) surgical interventions, including knee arthroplasty in 5 studies [13,18,26,27,30] or high tibial osteotomy in 3 studies [7,31,32], (ii) disease progression in 5 studies [5,24,28,33,34], (iii) exercise-based interventions in 5 studies [12,17,21,22,25], and (iv) the use of lateral wedge insoles in 5 studies [9,20,23,67,68].

Cross-sectional designs primarily assessed VT in the following contexts: (i) comparisons between adults with knee OA and asymptomatic controls in 9 studies [11,40,46,47,52,54,56,57,64], (ii) comparisons across OA severities in 10 studies [6,8,10,35,39,48,52,59,62,64], (iii) comparisons between sexes in 1 study [60], (iv) associations with alignment or joint structure in 10 studies [6,36,44,[46], [47], [48],^51^,^53^,^58^,^66^], (v) associations with pain or symptoms in 8 studies [8,[42], [43], [44], [45],^51^,^55^,^59^], (vi) associations with other gait metrics in 4 studies [37,41,49,59], and (vii) associations with muscle strength or coordination in 4 studies [39,40,61,65].

Additionally, 4 studies directly assessed the agreement of VT measurements across systems, comparing inertial sensors to motion capture [38,57] or visual assessment [62,63]. One study also examined the reliability of VT measurement obtained using inertial sensors [56].

### 3.2 Visual assessment

**Fig. 4:**
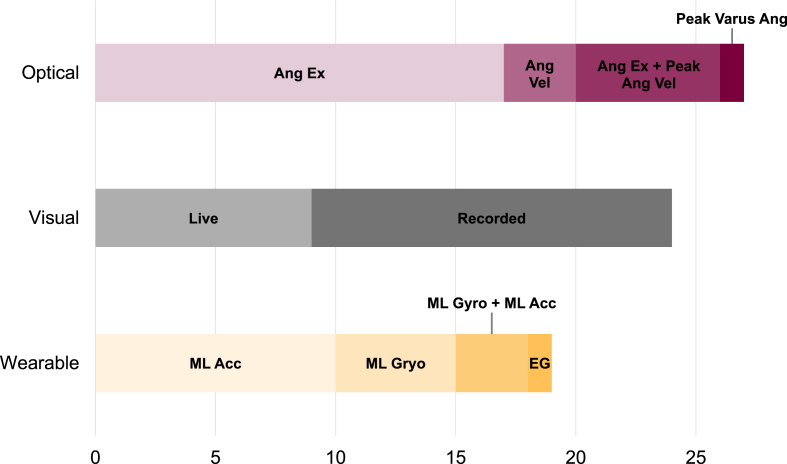
Tally of methods used to quantify varus thrust based on assessment modality. ML ​= ​mediolateral; Acc ​= ​acceleration; EG ​= ​electric goniometer; Ang ​= ​angular; Ex ​= ​excursion; Vel ​= ​velocity; Gyro ​= ​gyroscope.

A total of 24 studies assessed VT visually in 10,797 adults with knee OA. This was typically done by one or more rater determining the presence or absence of VT either from video recordings in 15 studies [7,17,19,21,22,28,33,34,[42], [43], [44], [45],^51^,^58^,^59^] or through live observation in 9 studies [5,10,35,37,62,63,67,69,70] (Fig. 4).

The most common operational definition described VT as a “dynamic worsening or abrupt onset of varus alignment during the weight-acceptance phase of the gait cycle, with a return to more neutral alignment during the lift-off and swing phases of the gait cycle” [34]. In many studies, VT presence was graded on a Likert-type scale. For example, Wink et al. [34] rated VT as “definitely present,” “probably present,” “probably absent,” or “definitely absent” across predefined proportions of steps. Participants were then classified as having gait with VT (*n* ​= ​301) or without VT (*n* ​= ​706) based on whether VT was “definitely present” during any steps (≥1) or “probably present” during “all steps.”

**Fig. 5:**
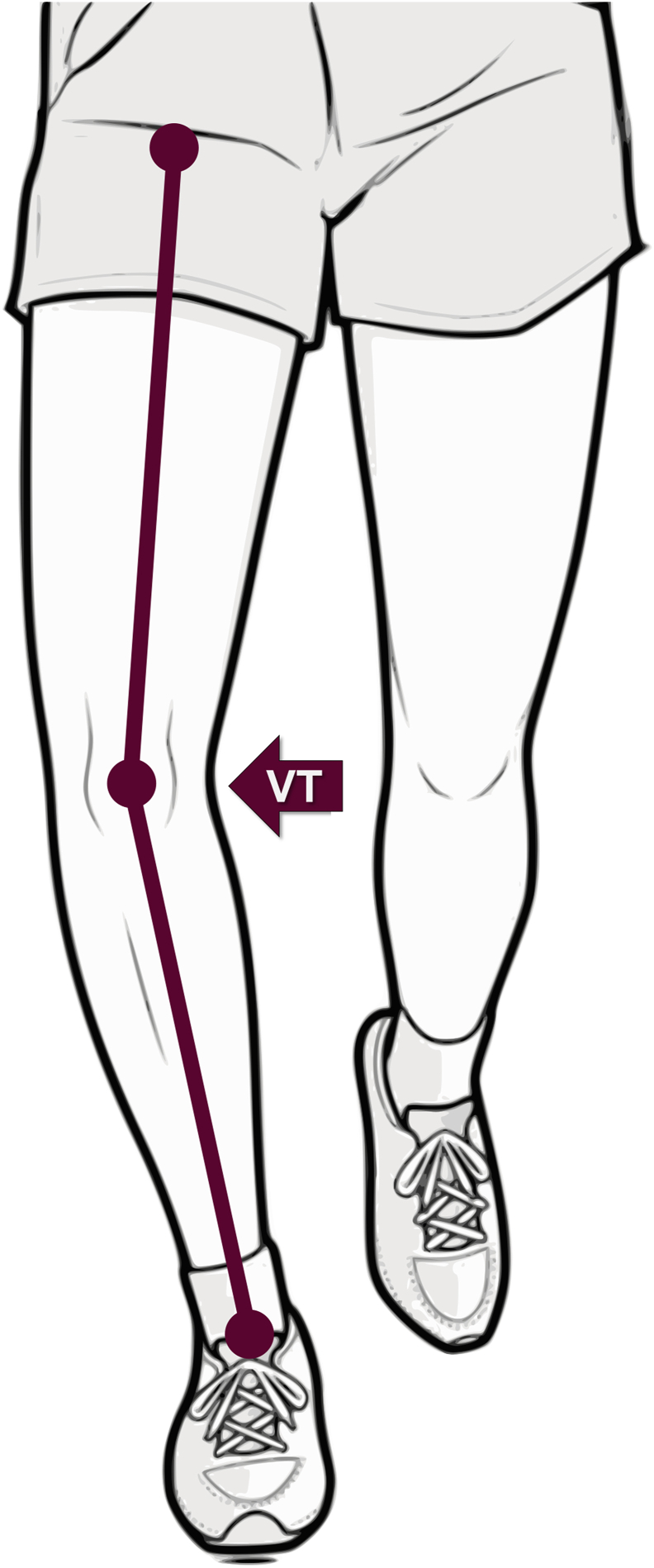
Representation of the presence of visual varus thrust (VT) during the loading phase of the gait cycle.

Additional definitions and classification schemes used in visual assessments are summarized in Table 1. A visual example of VT observed during the stance phase is provided in Fig. 5.

### 3.3 Optical motion capture assessment

**Fig. 6:**
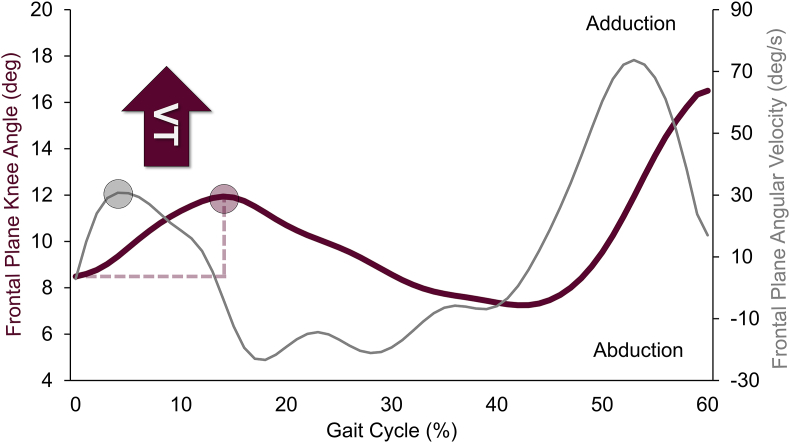
Representative plot of signals derived from optical motion capture for quantitatively estimating varus thrust (VT) during the gait cycle. The red and gray curves represent knee adduction and abduction angle or angular velocity, respectively. VT is commonly extracted, as indicated by the red and gray circles, representing peak knee adduction angle and peak angular velocity, respectively, during the loading phase. The red dashed line (0–15 ​% of gait cycle) indicates varus excursion and represents another quantitative method of estimating VT. (For interpretation of the references to colour in this figure legend, the reader is referred to the Web version of this article.)

A total of 27 studies assessed VT using optical motion capture in 1639 adults with knee OA. These studies aimed to quantify the largest frontal plane motion of the knee joint, using a range of approaches (Fig. 4). The most common method involved calculating frontal plane joint angle excursion from initial foot contact to a point later in stance in 26 studies [5,6,8,[11], [12], [13],^18^,[24], [25], [26],^29^,^30^,[36], [37], [38], [39], [40], [41],^46^,^47^,^50^,^52^,^54^,^60^,^65^,^67^]. However, the time window for this excursion varied across studies, from the first 10 ​% of the gait cycle [18,25,30,54], to a broader early stance phase [29,36,46,47], to the entire stance period [24,50,52]. A subset of these excursion-based studies [11,13,26,60] further required the presence of a peak angular velocity between the minimum and maximum angles used to determine the frontal plane excursion. Other studies [8,38,40,57] used this peak frontal plane angular velocity as a separate VT measure. A representative plot adapted from Sosdian et al. [11] illustrates these two approaches (Fig. 6), while Table 1 provides study-specific details on these methods.

### 3.4 Inertial sensor assessment

**Fig. 7:**
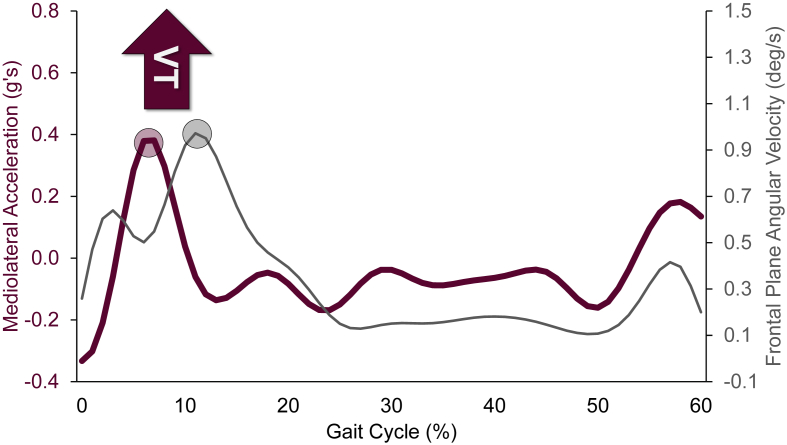
Representative plot of signals derived from an inertial sensor system for quantitatively estimating varus thrust (VT) during the gait cycle. The red and gray curves represent mediolateral knee acceleration and frontal plane knee angular velocity, respectively. VT is extracted, as indicated by the red and gray circles, representing peak knee lateral acceleration and peak angular knee adduction velocity, respectively, during the loading phase. (For interpretation of the references to colour in this figure legend, the reader is referred to the Web version of this article.)

A total of 18 studies used wearable inertial sensors to examine VT in 576 adults with knee OA [9,20,23,27,32,38,48,49,53,[55], [56], [57],[61], [62], [63], [64],^66^,^68^]. An additional study employed a six-degree of freedom electrogoniometer [31] (Fig. 4). The most common inertial-based VT metric was the maximum lateral acceleration of the tibia in 9 studies: [9,20,23,48,49,56,61,64,66], followed by peak tibia adduction angular velocity in 6 studies [27,38,56,57,62,64]. A representative plot, adapted from Ishii et al. [48] of the lateral acceleration and angular velocity methods is shown in Fig. 7. While all studies derived VT using either maximum lateral acceleration or peak adduction angular velocity, there was considerable variation in signal processing methods. Sensor placement also varied, though the most common location was near the tibial tuberosity (i.e., directly on, just proximal, medial, or on the fibular head), reported in 12 studies [9,20,23,27,48,49,55,56,61,62,64,68]. See Table 1 for additional methodological details.

## 4 Discussion

This scoping review is the first to comprehensively examine how VT has been defined, assessed, and applied in knee osteoarthritis research. We identified 63 studies that directly assessed VT in adults with knee OA, exploring its relationship to disease severity and progression, surgical and exercise interventions, alignment, and symptoms. Although VT may represent an accessible biomechanical marker, the diversity of assessment methods, along with limited validation, reliability testing, and methodological agreement, continues to hinder its broader adoption as a clinically meaningful gait metric in this population.

### 4.1 Visual assessment of varus thrust in research

Visual assessment remains a foundational approach in VT research, offering a practical means of identifying VT without the need for specialized equipment. Our review identified 24 studies using visual methods in over 10,000 adults with knee OA, many of which were drawn from three major longitudinal cohorts: the Mechanical Factors in Arthritis of the Knee study [5,37], the Osteoarthritis Initiative [10,28], and the Multicenter Osteoarthritis Study [33,34]. While overlap across these cohorts introduces some redundancy, they have provided valuable insights into VT's association with disease progression. Specifically, a meta-analysis by D'Souza et al. [71] of three studies identified in the current review [5,28,34] reported a combined odds ratio of 1.97 for progression in knees with visual VT. However, it is important to caution that these findings stem from secondary analyses of data, with overlapping authorship. Complementary cross-sectional studies have also reported associations between visual VT and OA severity [10,35,59], as well as pain, symptoms, and other gait characteristics [42,44,45]. As summarized in Fig. 3, this body of work supports the potential of visual VT as a simple, clinically relevant proxy for joint loading, with links to both symptoms and disease progression. However, the visual assessment of VT remains inherently subjective, with considerable variability in protocols and interpretation across studies.

Across the literature, visual VT is most commonly defined using the criteria proposed by Chang et al. [5]: “the abrupt first appearance of varus (or the abrupt worsening of existing varus) while the limb is bearing weight during ambulation, with return to a less varus alignment during the non-weight-bearing (swing) phase of gait” (pg. 3897). This definition is typically interpreted as a binary classification, determined by one or more rater observing gait either live or via video recordings. Later studies introduced Likert-type scales (e.g., “definitely,” “probably,” or “definitely not” present) and additional ratings for rater confidence or step-to-step consistency [37,63]. However, these refinements are often collapsed into dichotomous groupings, typically “with” or “without” definite VT, thereby simplifying interpretation but potentially limiting their added value [21,34,45,51].

Despite its practicality, visual VT assessment suffers from methodological inconsistencies that undermine its reliability and clinical applicability. While the conceptual definition is relatively consistent, the operational definitions (i.e., how VT is measured and rated) vary considerably across studies, complicating cross-study comparisons. Moreover, reliability is inconsistently reported, and when available, shows substantial variability for both intra-rater (k ​= ​0.73–0.92) [5,34,42] and inter-rater reliability (k ​= ​0.73–0.75) [17,42]. Studies also differ in the number of raters, the extent of rater training, and consensus procedures, some employing a third reviewer [17,45], others relying on discussion [51]. As a result, while visual VT remains a promising and accessible marker of OA severity and progression, its broader utility and interpretation are constrained by inconsistent operationalization, limited protocol standardization, and the fact that many findings originate from overlapping datasets and research groups.

### 4.2 Optical motion capture assessment of varus thrust in research

Optical motion capture offers the potential for a more consistent and quantifiable assessment of VT, though it is more time- and resource-intensive to implement. While it has been used in more studies than visual assessment (27 vs. 24), the total number of participants assessed with motion capture is markedly lower (1639 vs. 10,797), reflecting its logistical constraints. Despite the precision of this approach, definitions and protocols for quantifying VT using motion capture remain highly variable. Most commonly, VT has been defined as frontal plane knee joint excursion (See Fig. 4 and Table 1), though some studies have used peak frontal plane angular velocity or even simply the peak adduction angle [[11], [12], [13],^60^]. Like visual VT, motion capture-derived VT has been associated with disease severity, pain, symptoms, and functional outcomes [40,65]. Notably, optical motion capture has been used more often than visual assessment in longitudinal studies of post-surgical outcomes, despite its higher demands on equipment and setup (see Fig. 3).

In surgical studies, VT has been explored as a marker of recovery and dynamic knee mechanics following procedures such as high tibial osteotomy and total knee arthroplasty using motion capture. For example, Deie et al. [18] found that VT reductions were sustained only after open wedge osteotomy, while improvements following the closed wedge technique diminished by six months, highlighting VT's sensitivity in detecting subtle differences in surgical outcomes. In contrast, Paterson et al. [13,26] reported no change in VT at either six months or two years post-operatively, despite reductions in the peak knee adduction moment at both time points. However, comparing these findings is challenging, not only due to the distinct nature of the surgical procedures but also because of the variability in VT measurement methods. This inconsistency, typical across VT research, complicates drawing broader conceptual conclusions and underscores the need for standardized assessment protocols.

While optical motion capture enables detailed joint-level kinematic assessment, the wide variability in how VT is defined and processed across studies presents a major barrier to synthesis and comparison. Different metrics, including frontal plane excursion, peak angular velocity, and even peak varus angle, have all been used to characterize VT (Fig. 4), yet even within these categories, there is considerable variation in how the metrics are derived. As outlined in Table 1, studies differ in the portion of the gait cycle used, whether excursion or velocity (or both) are analyzed, and how thresholds are applied, all often with limited justification or reporting of psychometric properties. For instance, only one study has examined the reliability of optical VT measurement, reporting excellent intraclass correlations (ICC ​= ​0.98–0.99) for peak varus/valgus angles [37]. This methodological inconsistency, compounded by small samples and a reliance on secondary analyses, raises concerns about potential publication bias favoring significant findings. Moving forward, consensus on standardized definitions and consistent identification of gait events within kinematic signals is essential to improve reproducibility and clinical relevance. Establishing such protocols, alongside rigorous reliability testing, would enable the development of normative benchmarks, support longitudinal tracking, and advance efforts toward clinical thresholds for VT detection and monitoring.

### 4.3 Inertial sensor assessment of varus thrust in research

Compared with optical motion capture, wearable inertial sensors have emerged as a promising alternative for assessing VT due to their portability, affordability, and ability to be used in both clinical and real-world environments. The broader use of wearables for analyzing knee OA gait has grown in recent years [72], and this trend is reflected in VT research as well. Aside from an early study by Ogata et al. [9], all other investigations using IMUs to assess VT have been published since 2020, highlighting a substantial and recent surge in this area (Fig. 2). Despite their growing appeal, the number of studies using IMUs remain limited, with just 18 identified papers and a combined sample of 576 participants, the smallest of the three measurement modalities reviewed. Peak mediolateral accelerations and frontal plane angular velocities from sensors placed near the knee were the most common metrics used to quantify VT (Fig. 4). However, as with other approaches, both the specific applications and analytic methods varied considerably across studies.

Most inertial sensor studies on VT have been small, cross-sectional investigations, offering limited insight into progression or intervention effects. Common applications include distinguishing VT between individuals with and without knee OA or across OA severities, with metrics such as peak lateral acceleration and angular velocity showing significant group differences [48,64]. VT has also been associated with related clinical features such as medial meniscus extrusion and patient-reported outcomes [48,55]. A smaller number of recent studies have adopted more rigorous designs, including quasi-experimental interventions using insoles [23,68] and prospective evaluations following surgery [27,32]. Unlike those in the optical motion capture section, these studies observed reductions in VT post-operatively, particularly when accounting for walking speed, an often-overlooked covariate. Notably, Tsukamoto et al. [63] proposed a diagnostic threshold (<28.1°/s) for ruling out VT based on angular velocity, which was subsequently used in surgical evaluations by Sato et al. [27]. While this offers preliminary support for using IMU-derived VT in clinical decision-making, establishing a single threshold to dichotomize this complex and variable phenomenon, particularly across diverse IMU methodologies, remains unlikely.

As mentioned, the most commonly extracted VT metrics include peak lateral acceleration [20,48,64] and peak tibial frontal angular velocity during early stance [27,38,64]. However, the methods used to derive these measures vary considerably. Some studies define VT within the first 10 ​% of stance [48], while others extend the window to 20–30 ​% [62,64]. Others quantify VT using peak-to-peak differences in mediolateral acceleration [49], while only a few consider broader signal magnitude or acceleration range across stance [56,66]. While similar challenges exist in optical and visual methods, the inconsistency appears more pronounced in inertial sensor studies. This lack of consensus on signal processing and event definitions limits comparability between studies and impedes broader interpretation. Establishing clear guidelines and standardized processing protocols is essential to improve reproducibility and support the clinical translation of inertial-sensor-based VT assessment.

### 4.4 Current gaps and future directions

Despite increasing research on VT using visual observation, optical motion capture, and inertial systems, several critical gaps remain. While VT is generally understood as an abrupt varus or lateral knee motion during weight acceptance, how best to quantify this across modalities remains unclear. Visual VT, though limited by its semi-quantitative nature, remains a viable option when instrumentation is unavailable. There has been a growing push toward standardizing its assessment using Likert-type scales (e.g., “definitely present,” “probably present,” etc.) [37]. However, studies vary in how these ratings are dichotomized into presence or absence of VT. We recommend that future studies adopt the standardized Likert scale and report both the full categorical ratings and the binary classifications, with “definitely present” as a consistent cut-point.

For motion capture and inertial systems, the commonly extracted VT metrics presented in Fig. 4 provide a strong starting point for standardization. However, the lack of psychometric data on the reliability of these metrics limits the ability to make strong recommendations. Moreover, given that knee OA involves three-dimensional joint changes, the manifestation of VT in the frontal plane may be diminished or variable depending on OA severity and leg alignment, further complicating VTs detection and contributing to inconsistencies across studies. Previous gait analysis research suggest that frontal plane joint angles tend to be highly reliable for motion capture systems [37], and that peak lateral acceleration may demonstrate greater reliability than angular velocity in inertial systems [73]. Still, reliance on discrete peak-based measures may not fully reflect the underlying concept of VT. Future work should explore waveform-level analyses of frontal plane motion during stance, such as dimensionality reduction techniques (e.g., principal component analysis) previously applied to knee adduction moment data [74]. These approaches may better capture meaningful variability and support the development of robust, generalizable VT metrics.

Regardless of the specific VT metric selected, future research must adopt more rigorous and transparent methodological practices. This is particularly important in a field where no consensus exists on a standard approach, and many methods remain exploratory. Detailed reporting of preprocessing steps and signal extraction techniques is essential, not only for replicability, but also for understanding how methodological choices influence outcomes. Whether variability in VT metrics stems from insufficient methodological detail in prior literature, intentional innovation, or lack of awareness of previous approaches, future studies must clearly justify their analytic choices. Importantly, the field should shift toward more hypothesis-driven research with predefined expectations, rather than exploratory analyses that risk inflating positive findings. For example, only one study in the current review, Murro et al. [57], found no association between VT estimates from optical motion capture and IMUs, marking a rare null result in a body of literature that may be biased toward confirmatory outcomes. Strengthening transparency and methodological rigor, as demonstrated by Murro et al. [57], will help consolidate evidence rather than contribute to its current fragmentation. Finally, common confounders such as gait speed [32] and static alignment [5,45] should be more consistently accounted for to improve the interpretability of VT analyses.

### 4.5 Limitations

This review was conducted as a scoping review and, in alignment with its objectives, did not include a formal quality assessment or meta-analysis. The considerable heterogeneity in VT definitions, assessment protocols, and outcome measures across studies precluded meaningful synthesis through meta-analytic techniques. However, beyond the visual progression studies included in a separate review (e.g., D'Souza et al. [71]), few areas showed sufficient consistency to support formal synthesis, and the need to address methodological inconsistencies remains a more pressing priority. This review also focused specifically on VT during level walking gait and did not include studies examining VT during other functional tasks such as balance, weight transfer, or turning, contexts in which VT-like motion may also be relevant [75]. Additionally, this review was not able to clarify the interplay between static alignment and dynamic instability, an important but underexplored aspect of VT assessment. Finally, while a comprehensive search was conducted across multiple databases and updated through July 2025, it is possible that relevant studies indexed outside these sources or published in languages other than English were not captured.

## 5 Conclusion

This review highlights the increasing efforts to characterize VT during gait and the emergence of multiple assessment modalities, each with distinct strengths and limitations. While visual assessment remains widely used and accessible, it lacks standardization and reliability. Optical motion capture offers detailed kinematics but suffers from inconsistent definitions and high resource demands, while inertial sensors provide a promising, scalable alternative, though current methods remain variable and under-validated. Moving forward, the development and adoption of standardized, psychometrically sound VT metrics, alongside transparent, hypothesis-driven research practices, will be critical for improving comparability across studies, supporting longitudinal monitoring, and advancing the clinical utility of VT in the management of knee osteoarthritis.

## Author contributions

Conception and design (DK); Analysis and interpretation of the data (VD, DK); Collection and assembly of data (VD, ZM, JK, MR, FG, JW, YA, EO, DK); Obtaining funding (VD, DK); Drafting of the article (VD, ZM, JK, MR, FG, JW, YA, EO, DK); Critical revision of the article (VD, DK). All authors have approved the final version.

## Declaration of competing interest

The authors declare no competing interests and no funding sources had any role in this research.
